# Correlative Chemical Imaging and Spatial Chemometrics Delineate Alzheimer Plaque Heterogeneity at High Spatial Resolution

**DOI:** 10.1021/jacsau.2c00492

**Published:** 2023-03-07

**Authors:** Patrick M. Wehrli, Junyue Ge, Wojciech Michno, Srinivas Koutarapu, Ambra Dreos, Durga Jha, Henrik Zetterberg, Kaj Blennow, Jörg Hanrieder

**Affiliations:** † Department of Psychiatry and Neurochemistry, Institute of Neuroscience and Physiology, Sahlgrenska Academy, University of Gothenburg, Mölndal 431 80, Sweden; ‡ Clinical Neurochemistry Laboratory, Sahlgrenska University Hospital Mölndal, Mölndal 431 80, Sweden; § Department of Neurodegenerative Disease, Queen Square Institute of Neurology, University College London, London WC1N 3BG, U.K.; ∥ U. K. Dementia Research Institute at University College London, London WC1N 3BG, U.K.; ⊥ Hong Kong Center for Neurodegenerative Diseases, Sha Tin, N.T. 1512-1518, Hong Kong, China

**Keywords:** mass spectrometry imaging (MSI), light microscopy (LM), matrix-assisted laser desorption/ionization (MALDI), spatial chemometrics, image analysis, correlative imaging, Alzheimer’s disease (AD), amyloid pathology

## Abstract

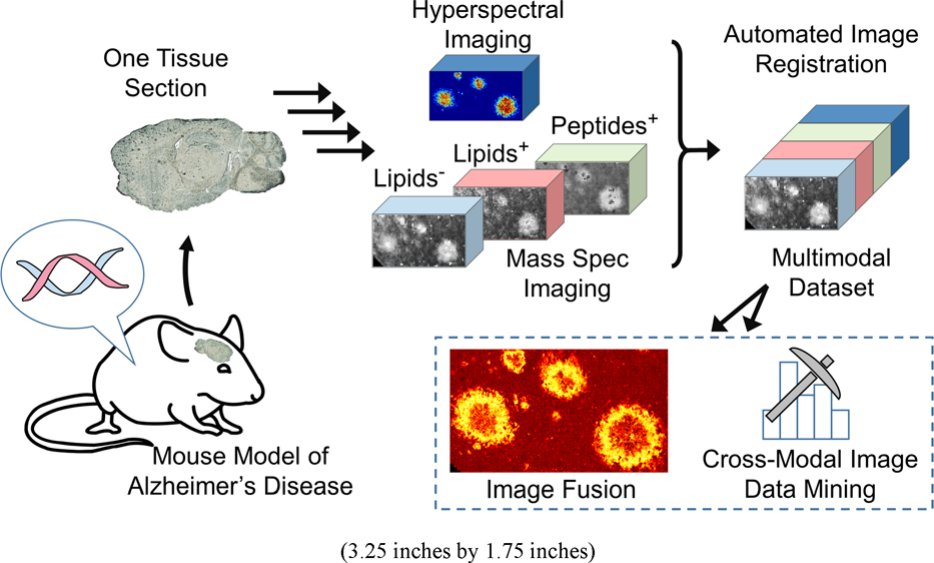

We present a novel, correlative chemical imaging strategy based on multimodal matrix-assisted laser desorption/ionization (MALDI) mass spectrometry imaging (MSI), hyperspectral microscopy, and spatial chemometrics. Our workflow overcomes challenges associated with correlative MSI data acquisition and alignment by implementing 1 + 1-evolutionary image registration for precise geometric alignment of multimodal imaging data and their integration in a common, truly multimodal imaging data matrix with maintained MSI resolution (10 μm). This enabled multivariate statistical modeling of multimodal imaging data using a novel multiblock orthogonal component analysis approach to identify covariations of biochemical signatures between and within imaging modalities at MSI pixel resolution. We demonstrate the method’s potential through its application toward delineating chemical traits of Alzheimer’s disease (AD) pathology. Here, trimodal MALDI MSI of transgenic AD mouse brain delineates beta-amyloid (Aβ) plaque-associated co-localization of lipids and Aβ peptides. Finally, we establish an improved image fusion approach for correlative MSI and functional fluorescence microscopy. This allowed for high spatial resolution (300 nm) prediction of correlative, multimodal MSI signatures toward distinct amyloid structures within single plaque features critically implicated in Aβ pathogenicity.

## Introduction

Over the last years, mass spectrometry imaging (MSI) has emerged as a powerful tool for chemical imaging to increase understanding of spatial biochemical distribution dynamics in tissue that are associated with histopathological processes.^1−7^ Moreover, acquisitions of multiple chemical imaging modalities contribute with complementary molecular information, specifically multimodal MSI or the integration of MSI with histological microscopy, vibrational spectroscopy, magnetic resonance imaging, as well as fluorescence microscopy.^8−13^ The acquisition of imaging data in multiple modalities yields datasets that may be spatially misaligned. In order to combine such datasets, the imaging data need to be registered to one another, meaning precisely geometrically aligned and image distortion-corrected.^14^ However, the registration of MSI images may be particularly difficult due to image noise and low contrast. Consequently, manual selection of accurate control points may be challenging, and machine-based registration approaches struggle to converge. While MSI data are commonly complemented with other modalities, cross-modal interpretation is often subject to human judgment. Workflows that incorporate image registration procedures are usually applied for co-representing images rather than to mine data across modalities.^15,16^

A major bottleneck is then the comprehensive statistical evaluation of multimodal imaging data. Here, statistics are typically performed on averaged data from assigned regions of interest (ROI) at the expense of spatial information.^17^ Elegant multivariate data analysis approaches have been pioneered integrating MSI data together with orthogonal imaging, typically histological microscopy, for comprehensive multimodal data analysis.^11,18−20^ These integrative multimodal analyses were, however, solely considering single ion mode MSI data. This highlights the need for both improved data processing workflows for accurate MSI image alignment and more powerful data mining strategies for the interpretation of multimodal data at the acquired MSI image resolution.^21−23^

The goal of the present work was, therefore, to leverage and significantly extend on previous multimodal imaging approaches including those developed in our lab,^8,10^ to provide a spatial strategy for comprehensive acquisition, registration, and integration of multimodal MSI and microscopy data to interrogate complex biological tissues while maintaining single pixel resolution. We developed new sample preparation workflows and imaging protocols that allow for trimodal matrix-assisted laser desorption/ionization (MALDI) MSI (negative ion mode lipids, positive ion mode lipids, and peptides) and interlaced fluorescence microscopy to be acquired on a single brain tissue section. We present a novel computational workflow that implements effective data processing and automated image registration and integration enabling multivariate statistical modeling of chemical information across multiple modalities while reducing human bias. Finally, we extend these analyses toward image data fusion to predict MSI ion distributions (10 μm) at microscopy image resolution (300 nm), while making use of hyperspectral microscopy information provided by using functional luminescent probes. We demonstrate the potential of the method toward delineating chemical traits of amyloid beta (Aβ) plaque pathology, the main pathological hallmark of Alzheimer’s disease (AD), in a transgenic mouse model (tgAPPSwe). We and others have been demonstrating the potential of MSI to interrogate Aβ pathology in human brain^24−31^ and mouse models.^8,32−39^ Herein, we demonstrate our novel chemical imaging strategy to identify multimodal imaging signatures associated with structurally heterogeneous Aβ plaque pathology at the micrometer scale. This is important as structural plaque heterogeneity has been associated with heterogeneous, clinical presentation of AD, such as cognitive performance, while the underlying chemical traits remain unclear.^40−43^

## Results

### Automated Alignment of Multimodal MSI Data

We here demonstrate a novel strategy for the acquisition, integration, and analysis of multimodal MSI data as well as functional fluorescent microscopy using structure-sensitive amyloid probes.^44^

For this, we generated two independent trimodal MSI datasets of cortical AD mouse brain that each incorporate positive and negative ion mode lipid data and peptide data [consisting of 673 (53%) variables in the negative ion mode lipid modality, 553 (43%) variables in the positive ion mode lipid modality, and 20 (2%) variables in the peptide modality].

The first step of this approach involves the acquisition of multimodal MSI data and their integration into a common, spatial data matrix that retains single pixel resolution.

MSI allows for acquisition of multimodal imaging data from the same tissue section. Commonly this involves the combination of MALDI-based metabolite and lipid imaging in positive and negative ion modes that are acquired sequentially on the same tissue without interruptions.^8^ This approach has been expanded toward different compounds by re-application of another more suited matrix or tissue washing depending on the molecular targets of interest (i.e., other lipids or peptides/proteins). Alternatively, this discontinuous multimodal imaging involves the combination of different MSI techniques such as secondary ion MS and MALDI^45^ or desorption ionization electrospray ionization and MALDI.^46^

For sequential analyses from the same preparation (dual polarity lipid MSI), the coordinate systems of the pixels in the two datasets correspond exactly and the data can be united into one multimodal dataset without the need for image registration.^47^ In contrast, discontinuously acquired multimodal MSI data are commonly spatially misaligned. Repeated measurement of the same tissue may be done with interruptions where additional tissue treatment steps are performed such as washing steps and matrix re-application for lipid and protein imaging, as performed here for both positive lipid and protein imaging. In such cases, the resulting MS imaging data will likely be spatially misaligned because of the tissue deforming during experimental procedures. Moreover, in certain cases, consecutive sections are required, and therefore image registration is necessary to achieve alignment of the corresponding pixels in each dataset.^22^ An accurate pixel correspondence between datasets is hence critical for downstream spatial chemometrics analyses.

Our first aim was, therefore, to establish an image registration workflow for precise alignment of imaging data acquired in different modalities. Image registration can be done using manual selection of fiducial points. This is, however, challenging on MSI data due to image noise and low contrast in single ion images. Consequently, it is difficult to achieve a registration accuracy suited for the investigation of small features such as Aβ plaques (50–100 μm) in AD pathology. The use of glass etched fiducials is not suitable, as they do not account for tissue deformations that can occur during experimental procedures. An automated method for the registration of MSI data based on a gradient descent algorithm has been previously presented.^48^ However, while gradient descent might work for image data with tissue edges, the alignment of small anatomical features without tissue edges was not successful for our data (no convergence). We have therefore developed a workflow for the registration of multimodal data employing an intensity-based optimization algorithm.

**Figure 1:**
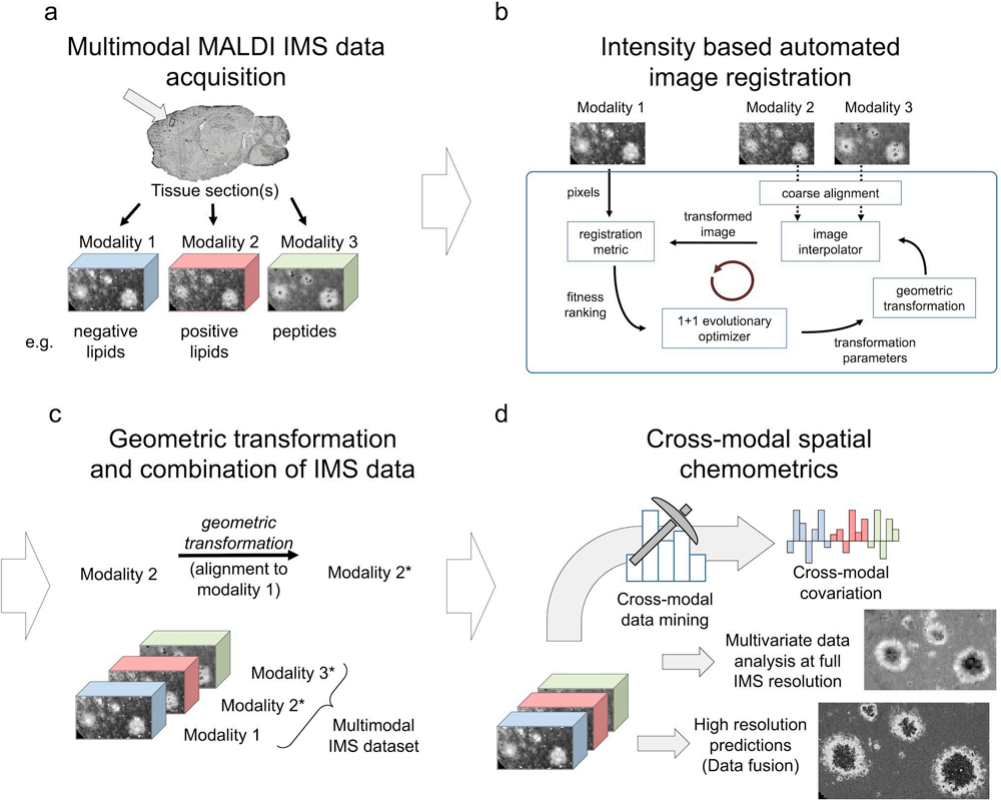
Overall workflow for the multimodal exploration of biological tissues by MALDI MSI and spatial chemometrics. (a) MALDI MS imaging of tissue sections in various modalities provides distribution maps of different biochemical species such as lipids and peptides. (b) Registration procedure for single pixel alignment is done using an intensity-based automated image registration approach.^50^ PCA score images are used as reference images for image registration due to their lower noise and higher contrast compared with single ion images.^49^ (c) Geometric transformation MSI datasets combined into one multimodal dataset. (d) Advanced spatial chemometrics analysis of the combined imaging data. This involves multivariate image analysis at the original image resolution without averaging and includes multiblock orthogonal component analysis, to extract covariations within and between modalities. High-resolution prediction of ion distribution by image fusion enhances histological interpretation.

Image registration procedures usually require one representative image of each modality, but MSI datasets consist of thousands of ion images which makes appropriate selection of references challenging. Moreover, registration of single ion images may be particularly difficult due to typical image noise and low contrast. To solve both of these issues, we made use of principal components analysis (PCA) to capture the variance in the MSI datasets, while separating noise, an approach that was previously presented where magnetic resonance imaging (MRI) and MSI data were registered.^49^ This approach produces a limited set of PCA score images, which are typically of higher contrast and less noisy than single ion images. From this set of images, a suitable reference for image registration can be selected. We selected reference images that represented prominent anatomical- or pathological features and, at the same time, were matching best between the modalities (Figure 1a). We then applied an intensity-based, automated image registration method to estimate the geometric transformation required to align the corresponding pixels between two reference images and MSI modalities, respectively (Figures 1b and S1). The method solves image registration problems through iterative optimization of a predefined dissimilarity metric using a 1 + 1-evolutionary optimizer approach.^50^ This optimizer is suitable for the registration of images with different brightness and contrast and, therefore, particularly suited for the registration of multimodal images. A further challenge is to estimate registration accuracy, which is not always straightforward, as spatial offset can not only be rigid but also be confounded by distortional and rotational factors (Figure S1a–f). We applied a combination of structural similarity, Jaccard similarity index, and mutual information (MI) metric as numerical evaluation of the automated image registration method (Figure S1a). Together, the 1 + 1-evolutionary optimizer approach was found to produce superior image registration results compared with manual registration by fiducial points (indicated by the smaller relative standard deviation of 0.036–0.138, Figure S1b).

While evolutionary optimizers aim at finding a global minimum, they are subject to non-optimal (local) minima, leading to poor convergence results. The 1 + 1-evolutionary strategy locally adjusts parameters to provide a mechanism to step out of non-optimal minima. Optimizer parameters need to be supplied to the algorithm and tuned for successful convergence. However, we found that tuning optimizer parameters manually is tedious, and we implemented an algorithmic exhaustive search between set parameter limits to maximize structural similarity between registered images.

While we present extensive registration efforts to align the pixel arrays of different modalities, we acknowledge that an uncertainty of pixel correspondence always remains. This uncertainty of pixel correspondence arises as a result of, for example, the offset between actual measurement locations and laser foci, which potentially vary slightly between modalities. Furthermore, interpolation of image data can introduce additional variation (such as blurring) that may confound accurate pixel correspondence. However, MSI datasets tend to exhibit positive spatial autocorrelation, which may lessen some of the impact of pixel correspondence uncertainty when multivariate modeling is applied (Figure S1). On this note, the use of imaging data obtained from consecutive tissue sections weakens the statistical pixel correspondence and should be avoided for image analysis purposes at full pixel resolution (Figure S1).

The resulting transformation matrix can then be applied to geometrically transform the MSI dataset (Figure 1c) and, finally, the registered data can be combined into one matrix and analyzed as one multimodal array for subsequent multivariate analysis(Figure 1d).

### Spatial Chemometrics Using OnPLS Modeling Reveals Covariation of Multi-Modal Chemical Signatures in Pathological Features of AD

Statistical analysis of MSI datasets is commonly done by averaging spectral data of multiple pixels from ROIs. While averaging pixel information reduces spatial information, it provides a method to analyze multimodal data without the need for image registration. A further reason for combing spatial information from different pixels would be to enhance mass spectra quality. Averaged mass spectra are typically less noisy compared to single-pixel spectra as S/N increases with sqrt(*n*), where *n* is the number of averaged spectra. This is especially useful with low abundant peaks in high spatial resolution data in which single-pixel spectra are often too noisy due to reduced MSI sensitivity and do not permit robust peak detection and quantification.

This ROI approach works for analyses of datasets where the full acquired spatial information is not critical, but manual ROI selection also introduces user bias by selection of size and location of ROI. This further may be confounded by chemical inter- and intra-feature heterogeneity as, for instance, observed for beta-amyloid plaques in AD that vary in size, shape, structural morphotype, and chemical content both within and across different plaques.^40,51^ Therefore, retaining the spatial information at single pixel resolution is essential for the investigation of systems with heterogeneous pathological features.

To address these issues, we present an alternative approach that allows for comprehensive interrogation of multimodal imaging data of heterogeneous tissue areas, while maintaining the full image resolution to reveal chemical co-localization patterns that covary within and between imaging modalities.

In multivariate analysis of MSI data, each pixel of the imaging dataset is treated as individual observations while retaining its spatial information (coordinates) during data analysis to be able to later reconstruct score results into component score images. Each of the score images in multivariate image analysis constitutes a component, where score values are represented by color. Meaning of the representation can be drawn from the corresponding loading vectors. In that way, PCA and hierarchical clustering have previously been used to extract biochemical characteristics of tissues and cell cultures from MS imaging data.^18,52,53^ These approaches are, however, limited toward generating models for single-modal^54^ but not multimodal data blocks. This results in issues with respect to different measurement scales, adequate normalization, and block-specific noise, limiting interpretation of these projections models for multimodal (multi-block) data.^55,56^

Therefore, more advanced multivariate analysis approaches that examine a multi-block data structure are necessary. Multi-block data integration methods, specifically O2PLS, and multi-block orthogonal component analysis based on the OnPLS algorithm^55,57−60^ are potentially suitable approaches to handle the challenges arising with multimodal MSI data. OnPLS is a descriptive modeling technique with a purpose to reveal the relationships between multiple blocks of data and an aim to enhance interpretability of the results.^55,59,60^ These algorithms allow to model the joint and unique variation between blocks of data in an unsupervised fashion and can avoid the previously described problems of different scales and block specific noise. OnPLS components can then be investigated using scores and loadings in the same way as single-block methods, such as PLS or PCA.

**Figure 2:**
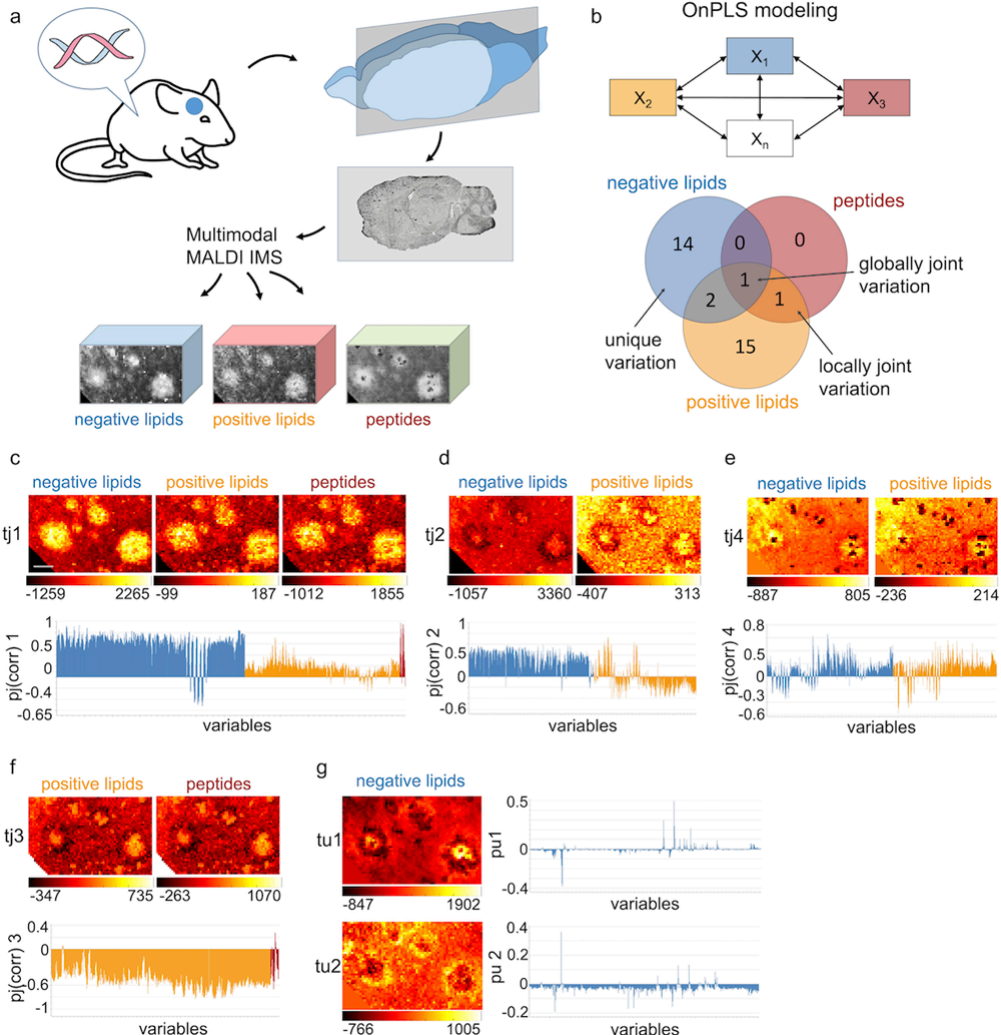
Spatial chemometrics analysis of multimodal MALDI MSI data of Alzheimer’s disease (AD) pathology. (a) Cortical brain tissues of atransgenic mouse model of AD (tgAPPSwe) is analyzed using MALDI MSI to obtain negative ion mode lipid-, positive ion mode lipid-, and peptide imaging data. (b) Unsupervised modeling using OnPLS allows for the analysis of relationships between all modalities. The Venn diagram shows partition of the variation within the three datasets where numbers refer to OnPLS components of globally joint, locally joint, and unique variation. (c) Globally joint component score image and loadings describing covariance among all three modalities. (d, e) Locally joint component of negative- and positive ion mode lipid data. (f) Locally joint component of positive ion mode lipid- and peptide data. (g) Unique variation in the negative ion mode lipid modality. Normalization: root mean square; scale bar = 100 μm.

We therefore used multiblock orthogonal component analysis, based on the OnPLS algorithm to interrogate our registered multimodal MSI data, to explore the relationships of the three modalities in an unsupervised manner (Figures 2a,b, S2, and S3).

Here, resulting globally joint component (tj1) images displayed the general, plaque specific covariation between all MSI modalities. Analytes increased or depleted within plaque structures relative to non-plaque areas are identified. The strongest globally joint correlations were found for LPE (18:0) (*m/z* 480.3, neg. lipids), LPC (16:0) (*m/z* 496.3, pos. lipids), and Aβ1–40 (*m/z* 4331, average mass, peptides), all previously found to localize to Aβ plaques (Figures 2c and S4a).^8,10^ In contrast, multiple lipid species showed a general plaque specific depletion pattern including sulfatides ST(d18:1_24:1) (*m/z* 888.6) and ST(d18:1_24:0(2OH)) (*m/z* 906.6) in negative lipid mode and PC(38:2) (*m/z* 852.6) in positive lipid mode (Figure S4a and Table S1).

A strength of multiblock OnPLS data analysis is to provide further locally joint components that provide the covariation also between just some modalities that can explain further detailed substructures. Specifically, locally joint covariation between positive and negative ion mode lipids species (tj2, tj4) captured, here, lipid localization patterns associated with structural plaque heterogeneity including peripheral and core specific localizations (Figures 2d,e and S4b,d). This exemplifies the strong advantage of using full imaging resolution together with multimodal data analysis with the potential to recognize detailed correlating localizations that could not be detected with univariate regression analyses. A second locally joint component (tj4) identified lipid core localizations with increased levels of PE-Cer(38:1) (*m/z* 715.6) and CerP(d36:1) (*m/z* 644.5) in negative ion mode, PE(P-34:3) (*m/z* 720.5) in positive ion mode, and decreased levels at the plaque core for LPI 18:0 (*m/z* 599.3), ST(d18:1_22:0 (*m/z* 862.6), and ST(d18:1_22:0(2OH)) (*m/z* 4 878.6) (tj4, Figures 2e and S4d).^61^ Moreover, positive lipid and peptide covariates emphasize plaque core localizations between all plaques detected. The plaque cores were found to consist of higher levels of Aβ(1–40) (*m/z* 4331), Aβ(1–40ox) (*m/z* 4347), and Aβ1–38 (*m/z* 4132) and were depleted in the majority of positive lipid species (tj3, Figures 2f and S4c).

Finally, multiblock component analysis also provides modality-unique components (tu) that capture variation specific for a modality. Here, in negative lipid data, unique variations (tu1 and tu2) revealed the core-localizing analytes PE-Cer 38:1 (*m/z* 715.5) and peripheral localization distributions for PI 38:4 (*m/z* 885.5) and PI 36:4 (*m/z* 857.5) around plaque structures (Figures 2g, S2c, S3c, and S4d).

In sum, multi-block modeling with its capability to detect cross-modal relationships allows for improved and unbiased biological understanding of the investigated systems. Visualization of scores from multivariate analyses together with corresponding loadings have the potential to reveal spatially defined chemical signatures that provide valuable new insights into the heterogeneous biocehmical distributions associated with plaque pathology.

### Image Fusion for Predictions of MSI-Derived Chemical Distributions at High, Microscopy-Level Resolution

Histological and immunohistochemical imaging using light microscopy (LM)-based methods offer a distinguished spatial resolution limited solely by the diffraction limit. MSI on the other hand is characterized by high molecular specificity and high content chemical information though at lower spatial resolution, particularly for peptide analysis.^62^ Data-driven image fusion combines the advantages of both techniques to produce image predictions that enhance histological interpretation and contribute to insight about complex biological systems at cellular length scales.^63^ Following the extensive cross (MSI) modality analysis, we aimed to extend the trimodal MSI approach toward integration with functional microscopy.

**Figure 3:**
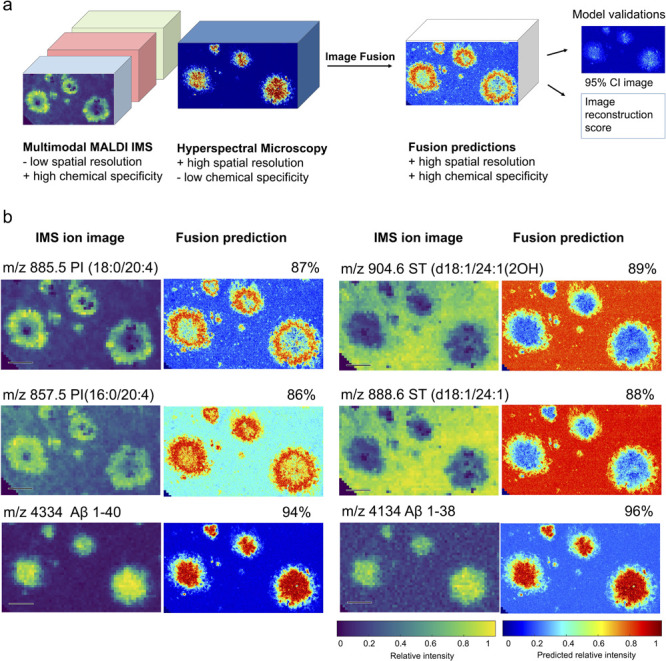
Data-driven image fusion: Prediction of molecular distribution at microscopy resolution. (a) Image fusion based on multimodal MALDI MSI and hyperspectral fluorescence imaging combining high chemical specificity of MSI with high spatial resolution of microscopy. (b) Single ion images and their fusion prediction at microscopy resolution. Reconstruction score in %. Scale bar: 100 μm.

For this, we applied data-driven image fusion based on multivariate linear regression^63^ to predict MSI ion distributions at high (LM) resolution. Specifically, we aimed to fuse the trimodal MSI data (positive/negative ion mode lipid and peptide data) with hyperspectral fluorescence amyloid imaging data obtained from the same biological tissue (Figures 3a and S5). To achieve this, we developed a tissue preparation workflow that allows for the acquisition of tetramodal imaging data on a single biological tissue minimizing artifacts and distortions. MALDI MSI acquisitions desorb analytes from the sample surface potentially impacting imaging modalities applied thereafter. The peptide analysis step of the previously described MSI3 workflow requires MALDI MSI acquisition settings that lead to laser ablation marks on the sample tissue. In order to avoid ablation artifacts, peptide analysis has to be performed last.^9^

For functional microscopy staining, we used structure-sensitive, fluorescent amyloid probes [luminescent conjugated oligothiophenes (LCO) dyes] that show differential binding to varying amyloid polymorphs and can be delineated with hyperspectral, fluorescence microscopy. The challenge with combining the LCO staining protocol with the MSI3 paradigm was to reconcile the order of acquisitions, matrix removal for LCO staining, and removal of the cover slip while preventing significant damage to the tissue.

The optimized MSI/LM acquisition workflow included sequential lipid MSI followed by LCO microscopy prior to final peptide MSI. One crucial step was to perform LCO staining prior to peptide MSI analysis. This produced fluorescence imaging data without tissue distortion and discernable laser ablation patterns, respectively (Figures S9 and S10). Image fusion calculation time increases with the number of MSI variables. Combining three MSI modalities can lead to very large datasets that comprise variables that are of lesser importance for fusion modeling and should be excluded. To do so, we applied a variable selection strategy based on variable importance in projection (VIP).^64^

The VIP value of each variable reflects its importance in the projection of the orthogonal projection to latent structure (OPLS) model, here between MSI (*x*-block) and microscopy (*y*-block), and provide a method to rank the variables.

In this case, the initial number of 2924 variables in the trimodal MSI dataset was reduced to 601 of the most important variables (VIP), reducing computational efforts. The data-driven fusion protocol reported by Van de Plas et al.^63^ incorporates automated means of filtering a large number of variables (i.e., ion species) and is restricted to single mode MSI data and histological staining. A previously presented approach for data-driven fusion^63^ incorporates automated means of filtering a large number of variables (i.e., ion species) and is restricted to single mode MSI data and histological staining. By passing pre-selected variables from VIP selection to the fusion protocol as demonstrated in our approach decreased the computation time from days to hours. (Further information on the VIP selection process and computation conditions can be found in the Supporting Information.)

Data-driven image fusion predicted high-resolution molecular distributions of various lipid and peptide species by modeling trimodal MSI data and hyperspectral fluorescence imaging data obtained from the same biological tissue section. The fusion results obtained with this approach yielded good reconstruction scores (Figures S6 and S7), well in alignment with previous studies.^63^ However, as with all predictive modeling, some uncertainty remains, and fusion predictions should be cross evaluated also with the acquired data of the corresponding ions. For this purpose, we display original, non-interpolated, ion images next to the fusion results (Figures 3b and S5–S7). Single MSI ion images and their fusion predictions at microscopy resolution supported by hyperspectral LCO microscopy reveal intricate details of amyloid polymorphism associated lipids and peptides. Specifically, phosphoinositol species PI(18:0_20:4) (*m/z* 885.5) and PI(16:0_20:4) (*m/z* 857.5) yielded high reconstruction scores for their predictions toward diffuse plaque periphery. In turn, Aβ1–38 and Aβ1–40 showed strong prediction scores across the entire plaque (Figure 3b). On the other hand, plaque-specific depletion of sulfatide ST(d18:1_24:1) (*m/z* 888.6) and its oxidized form ST(d18:1_24:1(2OH) (*m/z* 904.6) was observed, as suggested from the OnPLS models described above. The fusion predictions are evaluated together with 95% confidence intervals images displaying the confidence in the prediction in each pixel. There, locations with a narrow 95% confidence interval indicate where the confidence in the prediction is strong and, hence, whre there is strong support for the predicted ion intensity value (Figures S6 and S7).

## Discussion

In this study, we present a series of experimental and chemometric strategies to acquire and integrate multimodal molecular imaging data with different spatial resolutions. This comprises approaches for their alignment, registration, and combination into a common data matrix with maintained pixel resolution. This allowed for comprehensive interrogation of those multimodal data using novel, multivariate statistical modeling strategies to gain insight into disease pathology. We further make use of the multivariate results of these truly multimodal analyses for integration and prediction onto hyperspectral microscopy data providing functional insight of MSI signatures at scales not previously possible.

The accurate combination of multiple MSI modalities at single pixel resolution was enabled through precise image data alignment by an intensity-based automated image registration procedure.

For this, we employed an automatic image registration approach using an 1 + 1 evolutionary optimizer. The importance of image (data) alignment tools were recently surveyed in an extensive review by Balluff et al.^65^ As outlined, most approaches and software tools available implement interactive overlay methods or control point selection (used here as the manual method), which all result in user bias with variable image registration accuracies. Moreover, there is still no consensus on how image registration accuracy should be benchmarked and reported.

Herein, we provide therefore a selection of comparisons and metrics to demonstrate the relative accuracy to manual registration. This is a fair comparison given that most alignment approaches reported for the integration of MSI^n^ and MSI/LM data provide manual methods. Further, the relative standard deviations of all similarity metrics in the comparison were smaller for all automated alignments compared to manual ones indicating a more precise repeatability (relative standard deviation 0.036–0.138 smaller) of automated alignment (Figure S1).

Absolute accuracy measurements require a user input on both (ion- and light-) images for MSI/LM imaging data, which in turn contain user bias similar to control point selection for manual registration. Our methods for comparing accuracy of registration results are relative but without user bias overcoming this limitation.

This facilitated the generation of a trimodal MSI data matrix maintaining molecular information at single pixel resolution.

Although multivariate projection methods, such as PCA, can be used to model a single high-dimensional data block, they are theoretically not suited for modeling multiple data blocks simultaneously.^55^ Problems may arise due to different measurement scales and number of variables of the different data blocks generated for each modality. Also, combining multimodal data blocks into one matrix requires normalization to avoid variable-size biased projection models. Moreover, block-specific noise, such as measurement errors, could confound a projection model and make it difficult to effectively separate biologically relevant structures from noise.^55^

Finally, interpretation of projection models of multimodal data may be difficult, as they do not separate variation that is shared between data blocks and variation that is unique to each modality.^56^

OnPLS partitions the total variation into globally joint, locally joint, and unique parts. Global variation is shared between all data blocks of a multiblock dataset (Table S1), local variation is shared between at least two blocks, and unique variation occurs in only one data block. Decomposing levels of variation in this manner allow to identify also relatively small trends, in contrast to modeling methods that do not regard block structure and are, therefore, strongly biased toward large (global) variations.^55,59,60^

Unsupervised cross-modality chemometrics modeling of the combined data using multiblock orthogonal component analysis allowed for the identification of covariance structures and unique chemical variations at the acquired image resolution that otherwise could not be achieved with traditional methods and would be missed. The presented strategies, hence, lay the groundwork for correlative molecular imaging to further understand the interplay of underlying biochemistry with a multimodal imaging approach. Here, for example, LPE 18:0 and LPC 16:0 lipids that were associated with the dominant Aβ peptide 1–40 and 1–38, as contained in a common OnPLS component (tj1, Figures 2c and S4a), are likely directly involved in amyloid aggregation.

The number of joint components in OnPLS modeling is dependent on the covariation of the input datasets. If no covariation is found between two or more datasets, no joint components will be generated. Here, the peptide dataset contributed to few joint components only (Figure 2). A possible explanation for why not more components for the peptide modality were found may lie partly in the small fraction of peptide variables (2% of variables). However, this approach statistically links the different imaging modalities by the analytes’ covariance and colocalization. In our analyses, the imaging modalities were entirely congruent, meaning that there were no missing values, which is important as the OnPLS algorithm cannot process missing values.

However, modeling of systems with a large numbers of variables, and especially noisy variables such as the ones commonly observed in MSI, carries the risk of spurious correlations. As in any analysis, interpretation of multivariate models requires common sense. It is important to keep in mind that linear modeling methods, including OnPLS, are not suitable for detecting non-linear relationships, and in those cases, other methods should be used.^56^ Since not all variables were annotated, we acknowledge the uncertainty that not all variables within the lipid modalities are in fact lipids and could correspond to other low molecular weight compounds. Data analysis was performed with all data points from all peaks, that is, without spectral binning. Therefore, multiple loading variables may originate from the same species (isotopologues). We acknowledge that the mass accuracy of the used instrumentation limits the separation of isobars, which is however possible to resolve with recent advances within MALDI ion mobility spectrometry MSI.^51,66^

Reliable investigation of biological features of the scale as the observed core structures (30–40 μm) rely on the precise alignment of the modalities. This emphasizes the need for a method for accurate image alignment rather than averaging pixel information. On the other hand, image data that cannot be registered accurately, such as strongly distorted image data as well as data from intrinsically different consecutive sections, are likely not suited for this type of analysis.

We further developed a workflow that enable the acquisition of correlative multimodal imaging data combining both MSI3 and LM data. Specifically, we demonstrate the integration of OPLS modeling of trimodal MSI data with functional fluorescence microscopy imaging data to gain a deeper understanding of correlative relationships between MSI and fluorescence emission data that encode information on structural amyloid conformation. Impressive MSI-related fusion approaches have previously been presented for different multimodal imaging data such as for MSI with MRI^67^ and IHC^12^. Most prominently, the previously presented data-driven fusion algorithm for MSI data^63^ was based on PLS regression modeling and only MSI variables with strong relationships to the microscopy variables are considered for prediction. However, this approach is limited by the specificity of the histological imaging, in particular when interfaced with classic, morphological staining (such as H&E or Nissl staining), which are performed with brightfield microscopy and report only three channels (red, green, and blue). This restricts the prediction of distinct functional localizations that are not captured by the microscopy. For this reason, we expanded this approach toward both confocal microscopy and the use of multi-channel hyperspectral microscopy data that captures pathology-relevant variation. This allows for data-driven image fusion and prediction of multimodal MSI derived chemical imaging distribution at LM resolution along with structural information of amyloid aggregates. Here, even very small features that can be detected by microscopy but evade detection by MSI (due to limitations in MSI spatial resolution or sensitivity) are included in fusion predictions based on their hyperspectral signatures and their scoring in the regression model (Figure S8).

This allowed to identify amyloid maturation-specific chemical correlates across different MSI modalities within single plaques at single pixel resolution. This is critical as plaque maturation into structurally distinct plaque morphotypes has been associated with different trajectories of AD pathology across different forms and stages of AD^29,40^ While their spatial plaque association has been described before for most of the lipids detected here, those previous observations were on a more global, plaque ROI scale and mostly obtained at single mode MSI. The present approach provides more spatial and chemical details along with further validation of those lipids and their role in Aβ plaque pathology. Specifically, we identified phosphoinositol lipids to be associated with diffuse/premature aggregates, indicated by heptamer formyl thiophene acetic acid (h-FTAA) emission profiles prominently observed in the periphery of plaques. Interestingly, LPI has been identified as a ligand of TREM2, a microglial surface receptor critically involved in microglial activation and implicated in AD pathology by mechanisms of Aβ ingestion and seeding of Aβ plaque formation.^68−70^ The current approach provides the means to further explore lipid–Aβ interactions as well as the associated cellular environment at a more detailed picture.

The presented modeling and fusion approach can further be expanded to other multimodal imaging approaches including MSI^n^ and LM in combination with multiplexed IHC/mass cytometry.^71^ This will have great potential for other molecular histology applications such as tumor margin annotation and tumor classification.^72^

Together, the multimodal imaging and spatial chemometrics strategy described here is a pathway to deepen biological understanding through integration of molecular information from multiple imaging modalities, which is key to unlocking the full potential of multimodal imaging studies.

## Materials and Methods

### Chemicals and Reagents

All chemicals and solvents were used without further purification: acetic acid (Cat.#: 64197, VWR Chemicals), acetonitrile (ACN, Cat.#: 75058, Fisher Scientific), chloroform (Cat.#: 67663, LabScan), 1,5-diaminonaphthalene (DAN, Cat.#: 56451, Sigma Aldrich), 2′,5′-dihydroxyacetophenone (DHA, Cat.#: D107603, Sigma Aldrich), ethanol (Cat.#: V002075; Sigma Aldrich), formic acid (FA, Cat.#: 56302, Honeywell), and trifluoroacetic acid (TFA, Cat.#: 40967; Honeywell). LCO tetramer formyl thiophene acetic acid (q-FTAA) and heptamer formyl thiophene acetic acid were obtained from Prof. Peter Nilsson, Department of Chemistry, Linköping University. Water was obtained from a SynergyUV water purification system (Milli-Q, Merck Millipore).

### Animals and Tissue Preparation

Transgenic AD mice (number of animals: *n* = 2) carrying the Swedish mutation in APP (tgAPP_SWE_) were reared ad libitum at an animal facility at Uppsala University under a 12/12 light cycle. Fresh brain tissue samples were obtained from female, 18-month-old C57BL/6 mice. Animals were anesthetized with isoflurane and sacrificed by decapitation. The brains were dissected quickly with less than 3 min postmortem delay and frozen on dry ice. Animal procedures were approved by an ethical committee and performed in compliance with national and local animal care and use guidelines (DNr #5.8.18-20401/2020, Uppsala djurförsöksetiska nämnd). Frozen tissue sections (12 μm) were cut in a cryostat microtome (Leica CM 1520, Leica Biosystems, Nussloch, Germany) at −18 °C and collected on indium tin oxide conductive glass slides (Cat.#: 237001; Bruker Daltonics, Bremen, Germany) and stored at −80 °C. Prior to analysis, tissue sections were thawed under vacuum for 1 h.

### Matrix Application and MALDI MSI

For MALDI MSI of lipids, the DAN matrix was applied to unwashed tissue sections using a TM sprayer (HTX Technologies, Carrboro, NC, USA) combined with a HPLC pump (Dionex P-580, Sunnyvale, CA, USA). Before spraying, the solvent pump was purged with 70% aqueous ACN (ACN_aq_) at 300 μL/min for 5 min followed by manual rinse of matrix loading loop using a syringe. A matrix solution containing 20 mg/mL DAN in 70% ACN_aq_ was sprayed onto the tissue sections with the following instrumental parameters: nitrogen flow (10 psi), spray temperature (75 °C), nozzle height (40 mm), five passes with offsets and rotations, spray velocity (1250 mm/min), and isocratic flow of 50 μL/min using 70% ACN_aq_ as pushing solvent. After lipid analysis in negative ion mode, tissue sections were resprayed with three passes of the DAN matrix for lipid analysis in positive ion mode.

Hyperspectral imaging was performed between MALDI MSI lipid and peptide analyses on the same tissue section as described further below. Following hyperspectral imaging procedures, tissue sections were exposed to vapor of concentrated FA for 25 min for Aβ peptide signal enhancement, as previously described in detail.^10,29^ For MALDI MSI of amyloid peptides, DHA was used as matrix compound and applied using the TM Sprayer. A matrix solution of 15 mg/mL DHA in 70% ACN/2%CH_3_COOH/2%TFA was sprayed onto the tissue sections using the following instrumental parameters: nitrogen flow (10 psi), spray temperature (75 °C), nozzle height (40 mm), eight passes with offsets and rotations, spray velocity (1000 mm/min), and isocratic flow of 100 μL/min using 70% ACN as pushing solvent.

MALDI MSI was performed using a Bruker rapifleX TissueTyper TOF mass spectrometer (Bruker Daltonics), equipped with a Gaussian 355 nm Nd:YAG laser. Lipid analyses were performed in both positive- and negative ionization mode over a mass range of 400–2000 Da, whereby laser power settings were optimized for sensitivity in this mass range. Lipid imaging data were acquired at 10 μm spatial resolution, with the laser frequency of 10 kHz and 50 shots per pixel. External calibration was carried out using peptide calibration standard I (Bruker Daltonics). Peptide MSI data were acquired over a mass range of 1500–6000 Da, in linear positive ion mode, with 200 shots per pixel at a laser frequency of 10 kHz. The laser beam focus was set to “single” mode with beam scan, resulting in a lateral pixel resolution of 10 μm. External calibration was performed using Protein Calibration Mix 1 (Bruker Daltonics). Lipids and peptides were annotated by accurate mass following previous MS/MS based identifications reported by our group^35,51^ and others.^73,74^

### Fluorescent Staining and Hyperspectral Image Acquisition

The LCO staining procedure requires over-night incubation of the sample tissue with LCO dyes under a glass cover slip, which is critical for image quality. The exact preparation workflow for tetramodal imaging comprises (i) dual polar MALDI MSI of lipids with matrix re-application after the first acquisition, (ii) removal of matrix by washing, (iii) LCO fluorescence staining with cover slip mounted and over-night incubation, (iv) hyperspectral image acquisition, followed by (v) removal of cover slip for subsequent (vi) peptide MALDI MSI.

Fluorescent staining and hyperspectral imaging were performed after MALDI MSI lipid analyses and prior to MALDI MSI peptide analysis on the same tissue section. Therefore, the remaining matrix after MALDI MSI analysis was removed prior to fluorescent staining by sequential washes of 95% EtOH for 30 s, 95% EtOH for 60 s, 70% EtOH for 30 s, Carnoy’s solvent (60% EtOH, 30% chloroform, 10% acetic acid) for 90 s, and 95% EtOH for 10 s followed by 3 water washes of 2 min each. For the washing procedures, the glass slides were placed upright in fresh solvents and allowed to stand. The tissue sections were then incubated in dark at room temperature (23 °C) for 25 min with a combination of the LCO q-FTAA (3 μM in water) and h-FTAA (3 μM in water). After staining, the tissue sections were washed three times in water for 2 min each and mounted with a coverslip using Dako fluorescence mounting medium (Cat.#: S302380-2, Agilent Technologies) and incubated at room temperature (23 °C) for a minimum of 24 h before image acquisition.

Images were acquired on a Zeiss LSM-780 inverted confocal microscope equipped with a 32-channel GaAsP spectral detector using a Plan-Apochromat 20×/0.8 air objective and Zen Black software (Carl Zeiss, Jena, Germany). The excitation wavelength used was 458 nm with an average power of about 35 nW on the tissue sample. Acquisition was performed in lambda mode and using tile scan. A typical dataset comprised 32 spectral channels, covering wavelengths from 415 to 690 nm with 8.9 nm bandwidth, resulting in a *x*,*y*,λ-data cube.

Following hyperspectral imaging, coverslips were removed by soaking the glass slides in water for 24 h at room temperature (23 °C). Tissue sections were then subjected to sequential washes in water for 8 min, 70% EtOH for 60 s, and EtOH for 30 s and dried under vacuum before moving on to MALDI MSI peptide analysis. A comparison of different coverslip removal protocols and their effect on spectral quality can be found in Figure S9.

### Data Analysis

#### Data Processing

Data processing was performed in matlab R2020b with Bioinformatics Toolbox 4.14, Signal Processing Toolbox 8.4 and Image Processing Toolbox 11.1 (MathWorks, Inc.) installed. MALDI imaging data were exported from SCiLS Lab (version 2021c, Bruker Daltonics, Bremen, Germany) in the .imzML format and imported into matlab using the imzMLConverter by Race et al.^75^ Hyperspectral imaging data in the .czi format were loaded into matlab using the open-source matlab script “czi_spec_im_load” developed by Dr. Rafael Camacho (https://github.com/CamachoDejay/czi_spec_im_load). MS data were processed by baseline correction and normalization to root mean square peak picking. For our peak picking routine, we removed the noise between peaks and extracted all data points over the peaks without binning. Here, no peak convolution was done to allow for detection of potentially overlapping species. For the extraction of data for ROI analysis, the ROI was selected using the imageSegmenter() function (Image Processing Toolbox) to then subset the dataset to the ROI’s boundaries while retaining pixel coordinates.

### Image Data Registration

The spatial alignment (image registration) of MSI modalities was performed using in-house scripted matlab routines. Reference images for image registration were obtained through PCA of each modality as previously presented.^49^ The workflow for the alignment of MSI modalities involved a transformation matrix, which was estimated through automated image registration based on the PCA score images as reference images for each modality. The reference images were coarse-aligned by manual control point selection prior to the automated registration procedure. Control point selection was done using the cpselect() function (Image Processing Toolbox).

While the selection of three fiducial points would be sufficient for affine registration, we recommend select at least five points spread out over the tissue surface. More control points may improve initial registration, for example, when a control point is inaccurately selected. For the registration comparison, five control points were selected with even spread over the tissue surface at recognizable hallmarks in either image at variable locations between replicates (number of replicate alignment experiments: *N* = 5).

The automated image registration method utilized an intensity-based optimization approach particularly suited for multimodal applications. The optimization algorithm employed a 1 + 1-evolutionary optimizer with various settings paired with Mattes MI metric configuration.^76^ Thereby, the geometric transformation is estimated by minimizing the mean square error (MSE) between a fixed reference image (*I*, with *m* rows and *n* columns) and moving image (*K*) that is to be transformed (eq 1).
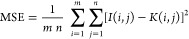
1

Optimizer parameters involving the search radius including growth factor, initial size, and minimal size were determined by algorithmic exhaustive search between set parameter limits to maximize structural similarity between reference images.^77^ We refer to Styner et al.^50^ for guidance in tuning optimizer parameters and setting parameters limits. Here, we describe the details about used optimizer settings and metric configurations, as well as parametric limits and step sizes of the exhaustive search. Mattes MI^76^ was kept constant, number of spatial samples = 500, number of histogram bins = 60, use all pixels = true. One-Plus-One Evolutionary optimizer settings presented as lower limit: increment: upper limit as applied in the exhaustive search: maximum iterations = 100 (constant); growth factor = 1.005:0.0005:1.1; ε = 1.5 × 10^–7^:1:1.5 × 10^–3^; initial radius = 6.25 × 10^–4^:1:6.25 × 10^–2^.

Finally, data cubes of MSI modalities were geometrically transformed using the imwarp function (Image Processing Toolbox) with bicubic interpolation. Bicubic interpolation was found to produce best results based on structural similarity, Jaccard similarity index, MI, and visual inspection of overlay comparisons.

### Jaccard Similarity Coefficient

The Jaccard index was calculated as the intersection of images A and B divided by the union of A and B, as shown in eq 2.^78^ For this, images to compare were converted to 8-bit unsigned integers before computing the Jaccard index using the jaccard() function in Matlab.
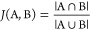
2

### Mutual Information

The MI of two images was calculated according to the axiom of information theory which is defined as
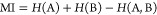
3

where *H*(A) and *H*(B) denote the individual entropies of image A and image B, and *H*(A,B) denotes their joint entropy. Since A and B are discrete images, entropies can be expressed as sums instead of integrals. Thus, their individual entropies can be calculated as
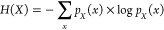
4where *p_X_*(*x*) is the probability distribution of pixels associated with image A or B.^79^ Probability distributions were computed by binning image values into histograms using accumarray() in Matlab. For this, images were converted to 8-bit unsigned integers and the floating point image values were assigned to unique IDs before passing the information to accumarray(). The joint entropy was calculated in a similar fashion
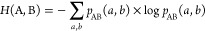
5

The joint entropy is minimized when the pixels in each of the images correspond exactly. In contrast, if the statistical relationship between the two images weakens, the joint entropy increases.^79^

A comparison of interpolation methods including nearest neighbor, linear, bilinear, and bicubic interpolation and details about the similarity metrics are outlined in Supplementary Results.

### Multivariate Modeling and Image Visualization

Registered image data were cropped to common area and concatenated along the spectral dimension into a multimodal dataset. For chemometrics analysis datasets were reshaped into a two-dimensional matrix where pixels are represented as rows and spectral vectors as columns. Pixels with a total ion current of zero (black pixels) from, for example, off-sample acquisition or pixels from outside irregularly shaped acquisition areas, were omitted from data analysis. Black pixel (bp) and data pixel (dp) matrices were extracted by logical indexing using indices to sums of the multimodal dataset (*X*_*i*, *j*_) that satisfy the equalities in eq 6. Thereby, the spectral values j of each individual pixel i were summed up.
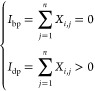
6

Multivariate analyses of MSI data were performed in SIMCA 17 (Sartorius Stedim Biotech, Umeå, Sweden) and comprised PCA and multiblock orthogonal projections to latent structures based on the OnPLS algorithm.^59,60^ OnPLS components describe variation in globally joint, locally joint, and unique parts for each data block as follows:
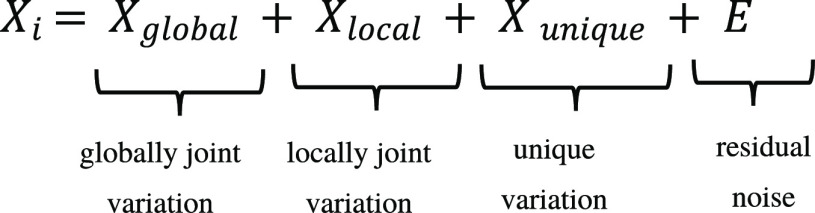
7

Thereby, global variation implies the structure that is shared between all blocks, local variation between at least two blocks, and unique variation that occurs in only one data block.^59,60^ Data were mean-centered for data mining (PCA and OnPLS of mass spectral data) and scaled to unit variance for predictive modeling (OPLS including hyperspectral channels). The number of evaluated components was based on the predictive performance as determined by the seven-block cross-validation of SIMCA. Component score matrices were transferred to matlab, where they were reunited with black pixels and reshaped into image dimensions prior to score image visualization. Respective loadings for the interpretation of the score images were generated in SIMCA software. Images were not interpolated beyond the spatial acquisition resolution for visualization purposes, and color scales were set by the default settings of Matlab.

### Variable Selection and Image Data Fusion

Variable selection for fusion predictions was based on predictive variable importance in projection (VIP) of OPLS models.^64,80^ To achieve this, hyperspectral image data were registered to the MSI data and geometrically transformed to then be incorporated as *Y* matrix in OPLS modeling. Variables of OPLS models with VIP values larger than 1 are the most relevant for explaining *Y*; therefore, variables with a VIP_OPLS-predictive_ > 1 were considered most important and selected for high-resolution prediction of ion distribution by image fusion.^80^ The VIP values were calculated using eq 8, where *K*_p_ is the total number of predictive variables, *A*_p_ is the total number of predictive components, and *P_a_* is the *a*^th^ component. The sum of squares (SS) has the subscript *comp* for the explained SS of *a*^th^ component and the subscript *cum* for the cumulative explained SS by all components in the model.
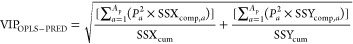
8

The sum of squares of all VIP’s is equal to the number of terms in the model hence the average VIP is equal to 1. Variables with VIP values larger than one, are the most relevant for explaining *Y*, therefore, variables with a VIP_OPLS-pred_ > 1 were considered most important and selected for further investigation and image fusion predictions.^64,80^

Data-driven image fusion was performed according to methods presented by Van de Plas et al.^63^ with changes. The image fusion models utilized partial least-squares regression to link MSI ion distributions to fluorescence imaging data. Fusion models were based on multimodality MSI variables of positive and negative ion mode lipid data and positive ion mode peptide data and hyperspectral fluorescence emissions from LCO staining. Only MSI variables with VIP_OPLS-predictive_ values greater than one were passed to fusion modeling. Fusion prediction images are evaluated as described in ref (63) and included reconstruction scores reporting deviation of prediction from measurement at MSI resolution. Further, absolute residual images and 95% confidence interval images were generated to provide location-specific prediction performance (Figure S10). While these measures of evaluation provide confidence in the fusion results, there is always some uncertainty left with predictive models. Therefore, we provide images of the original measurements of ion distribution to allow the fusion results to be cross-evaluated independently.

## Supporting Information Available

Supplementary results and additional data on image data alignment, OnPLS models, image fusion, and sample preparation (PDF)
Overview of the repeatability across different trimodal MSI datasets (XLSX)

## Notes

All source codes developed for this study are available on a GitHub repository at https://github.com/patrickwehrli/multimodal_ims.

## Notes

The datasets generated during and/or analyzed during the current study are not publicly available due to the large size (15.3GB) but are available from the corresponding author on reasonable request.

## Abbreviations

AD: Alzheimer’s disease
Aβ: beta-amyloid
DESI: desorption ionization electrospray ionization
LM: light microscopy
LCO: luminescent conjugated oligothiophenes
LPI: lysophosphatidylinositol
MALDI: matrix-assisted laser desorption/ionization
MS: mass spectrometry
MSI: mass spectrometry imaging
MVA: multivariate analysis
OnPLS: multiblock orthogonal projections to latent structures
PCA: principal components analysis
PE-Cer: ceramide phosphoethanolamine
PI: phosphatidylinositol
ROI: regions of interest
SIMS: secondary ion mass spectrometry
ST: sulfatide
VIP: variable influence on projection
