# Supplementary material for: Correlative Chemical Imaging and Spatial Chemometrics Delineate Alzheimer Plaque Heterogeneity at High Spatial Resolution
Source: JACS Au. 2023 Mar 7;3(3):762–74. doi: 10.1021/jacsau.2c00492 (PMC10052239; doi:10.1021/jacsau.2c00492)
Supplement: Supplementary file 1 — au2c00492_si_001.pdf [file au2c00492_si_001.pdf]

Supplemental Information

Correlative Chemical Imaging and Spatial Chemometrics Delineate Alzheimer Plaque Heterogeneity at High Spatial Resolution

Patrick M. Wehrli [a], Junyue Ge[a], Wojciech Michno[a], Srinivas Koutarapu[a], Ambra Dreos[a], Durga Jha[a], Henrik Zetterberg[abcde], Kaj Blennow[ab] and Jörg Hanrieder*[abc]

[a] Department of Psychiatry and Neurochemistry, Sahlgrenska Academy at the University of Gothenburg, Mölndal Hospital, House V, S-431 80 Mölndal, Sweden

[b] Clinical Neurochemistry Laboratory, Sahlgrenska University Hospital, Mölndal Hospital, House V, S-431 80 Mölndal, Sweden

[c] Department of Neurodegenerative Disease, Queen Square Institute of Neurology, University College London, London, United Kingdom

[d] UK Dementia Research Institute at University College London, London, United Kingdom

[e] Hong Kong Center for Neurodegenerative Diseases, Hong Kong, China

* Correspondence: Dr Jörg Hanrieder, E-mail: jh@gu.se

Content:

1. Supplemental Results
2. Supplemental Figures S1-10
3. Supplemental Table 1

1. Supplemental Results

Comparison of interpolation methods and registration accuracies

The accuracy of image registration results is frequently assessed using basic methods such as overlap scores, structural similarity score, and image difference.[1] Since these methods alone might not provide full evidence for accurate registration, we applied a combination of structural similarity, Jaccard similarity index, and mutual information metric as numerical evaluation and additionally confirmed registration by visual comparison via image overlay.[1-2]

In efforts of a controlled comparison of registration accuracies as well as suitable interpolation methods, we first unregistered a principal components analysis (PCA) scores image of a MALDI MSI dataset of mouse brain cerebellum to then register the distorted (unregistered) image to the original image. Various degrees of distortion were applied: (i) translational, (ii) translation and rotational (+4°), and (iii) translational, rotational and affine distortion. Image re-registration of distorted images was performed by a manual and an automated image registration approach. The manual image registration by fiducial point selection was done by selecting five control points at various locations spread over the tissue surface, in five replicate alignment experiments (N= 5). The same fiducial control points were then passed, as initial transformation matrix, to the automated image registration algorithm for on intensity-based optimization, providing a direct link between registrations. Registration accuracies were compared with various similarity metrics that were used in combination with visual inspection of overlay images: structural similarity, Jaccard similarity index, and mutual information. Similarity metrics are described in detail further below.

In all degrees of distortion, the automated method produced superior image registration results compared to manual registration by fiducial points (Figure S1b). Registration results appear very similar, therefore, structural similarity maps are presented to illustrate an exaggeration of inaccuracies (Figure S1c-f). It is important to note that rotational transformation and affine

distortion require interpolation and, therefore, re-registration of those images will unlikely reproduce the original image accurately. Furthermore, fiducial point selection between an image and its unregistered counterpart is straightforward compared to when images intrinsically differ as it is the case between modalities.

In this way, we further determined the best suited image interpolation method, which is required for the geometric transformation of image data. The comparison of interpolation methods included nearest neighbor, linear, bilinear, and bicubic interpolation. Bicubic interpolation was found to be the best suited interpolation method in the comparison based on the similarity metrics and visual observations of interpolation quality (Figure S1a). While nearest neighbor interpolation appears as higher scoring method based on Jaccard index and mutual information, structural similarity and visual comparison did not confirm this observation. Nearest neighbor interpolation created image artifacts that could explain the various metrics results.

2. Supplemental Information Figures

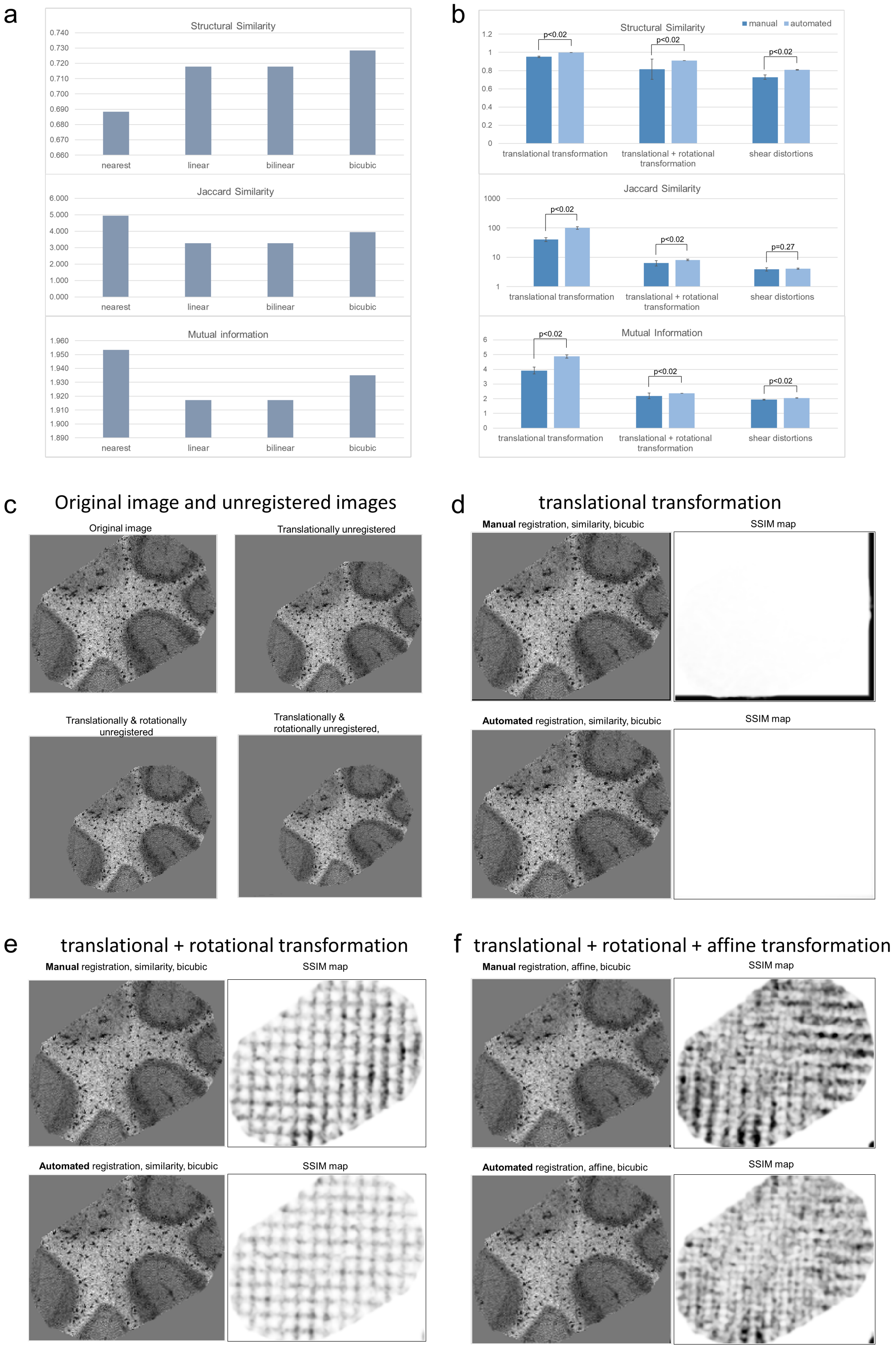

Supplemental Figure S1 Interpolation methods and registration accuracies. A principal components analysis (PCA) scores image of mouse brain cerebellum was geometrically transformed and then registered to the original image in efforts to compare interpolation methods and to compare registration performances between manual and automated image registration.

a) Comparison of interpolation methods by various similarity metrics shows bicubic and nearest interpolation methods to score highest. Visual inspection of the interpolated images using nearest interpolation revealed image artefacts while such were absent in bicubic interpolated images. Therefore, bicubic interpolation was preferred. Method naming refers to Matlab standard.

b) Comparison of manual and automated image registration accuracies for various degrees of geometric transformation. Error bars show standard deviation (number of technical replicates: N = 5). Automated registration scored higher on all similarity metrics evaluations and for all degrees of distortion except for Jaccard similarity of shear distortions. Relative standard deviations of similarity metrics were smaller for all automated comparisons:

rel. stdev	translational transformation		translational + rotational transformation		translational + rotational transformation + shear distortions	
	manual	automated	manual	automated	manual	automated
Structural Similarity	0.0087	0.0002	0.1380	0.0003	0.0330	0.0031
Jaccard index	0.1447	0.1092	0.2067	0.0564	0.1293	0.0419
Mutual information	0.0608	0.0237	0.0870	0.0007	0.0157	0.0061

c) Original PCA scores image of mouse brain cerebellum MSI data and after various degrees of image distortion (unregistering). These unregistered images were then used for various methods comparisons.

d) Visualization of image registration result of translational unregistered image and structural similarity (SSIM) map. The SSIM is exaggerating differences to the original image. Manual registration by control point selection (cps) shows dark areas on the SSIM map indicating differences between the registered and the original image while the automated registration method achieved a perfect match and is, therefore, fully white.

e) Visualization of image registration result of translational and rotational unregistered image and SSIM map. SSIM map of automated registration result contains less dark areas, than manual registration and is, therefore, superior. Due to interpolation during rotational unregistering a perfect match is unlikely to be achieved.

f) Visualization of image registration result of translational, rotational and affine unregistered image and SSIM map.

a

Blocks	Negative lipids	Positive lipids	Peptides
	R2X(cum)	R2X(cum)	R2X(cum)
**Joint components**	0.491	0.672	0.947
1	0.361	0.0359	0.875
2	0.461	0.224	--
3	--	0.609	0.947
4	0.491	0.672	--
**Unique components**	0.454	0.197	0
**Model total**	0.945	0.869	0.947

b

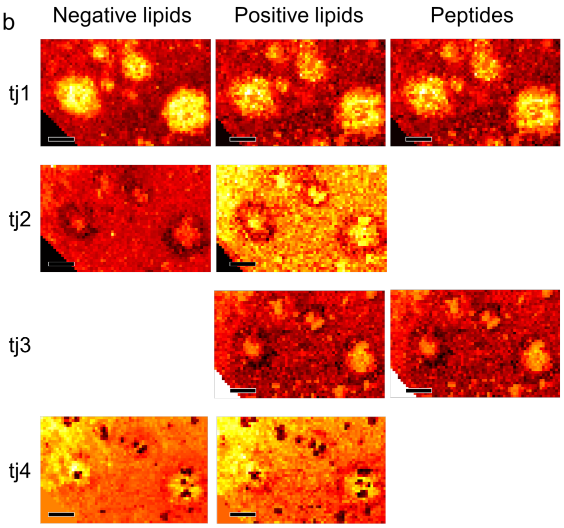

c

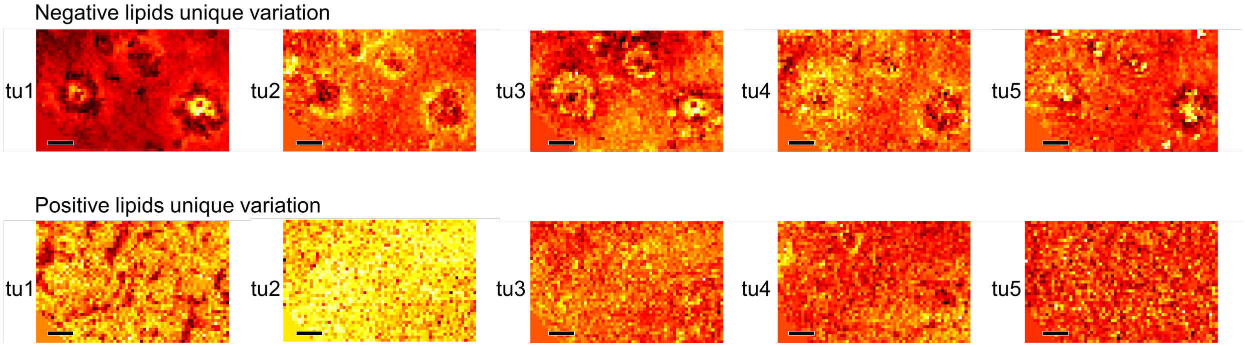

Supplemental Figure S2 OnPLS modeling details. a) Model information, fractions of cumulative modelled variation, b) joint component scores images, c) unique components of negative and positive ion mode lipids, no peptide unique components were generated. Scale bar: 100 µm.

a

Blocks	Negative lipids	Positive lipids	Peptides
	R2X(cum)	R2X(cum)	R2X(cum)
**Joint components**	0.809	0.448	0.939
1	0.564	0.0186	0.939
2	0.722	0.359	--
3	0.809	0.448	--
**Unique components (tu)**	0.14	0.385	0
**number of tu**	4	14	0
**Model total**	0.949	0.832	0.939

b

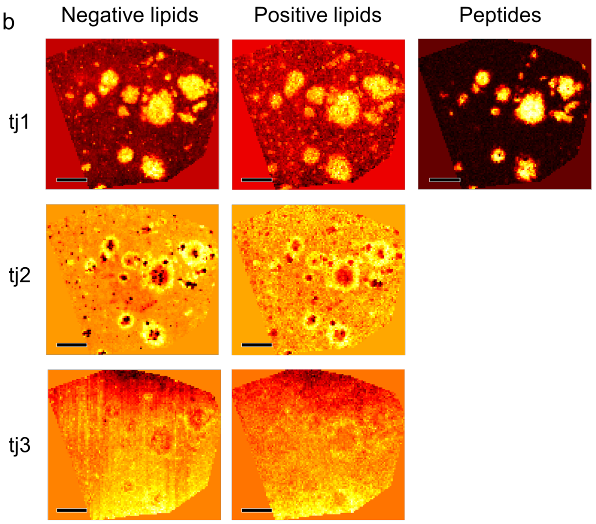

c

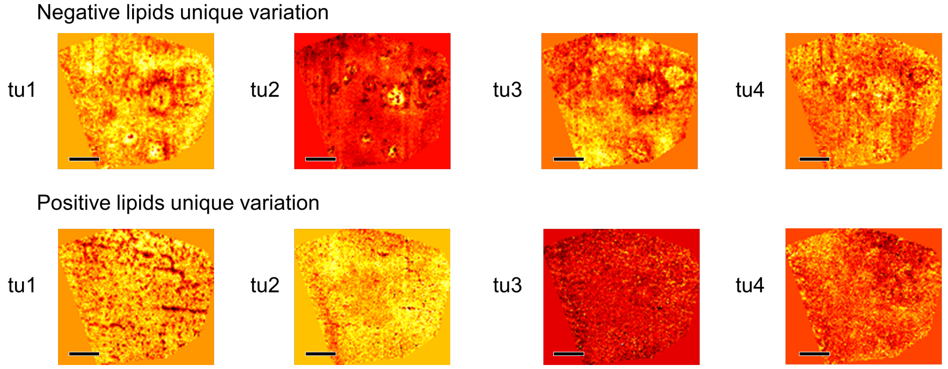

Supplemental Figure S3 OnPLS modeling of tgSwe mouse brain cortical tissue. a) Model information, fractions of cumulative modelled variation, b) joint component scores images, c) unique components of negative and positive ion mode lipids, no peptide unique components were generated. Scale bar: 200 µm.

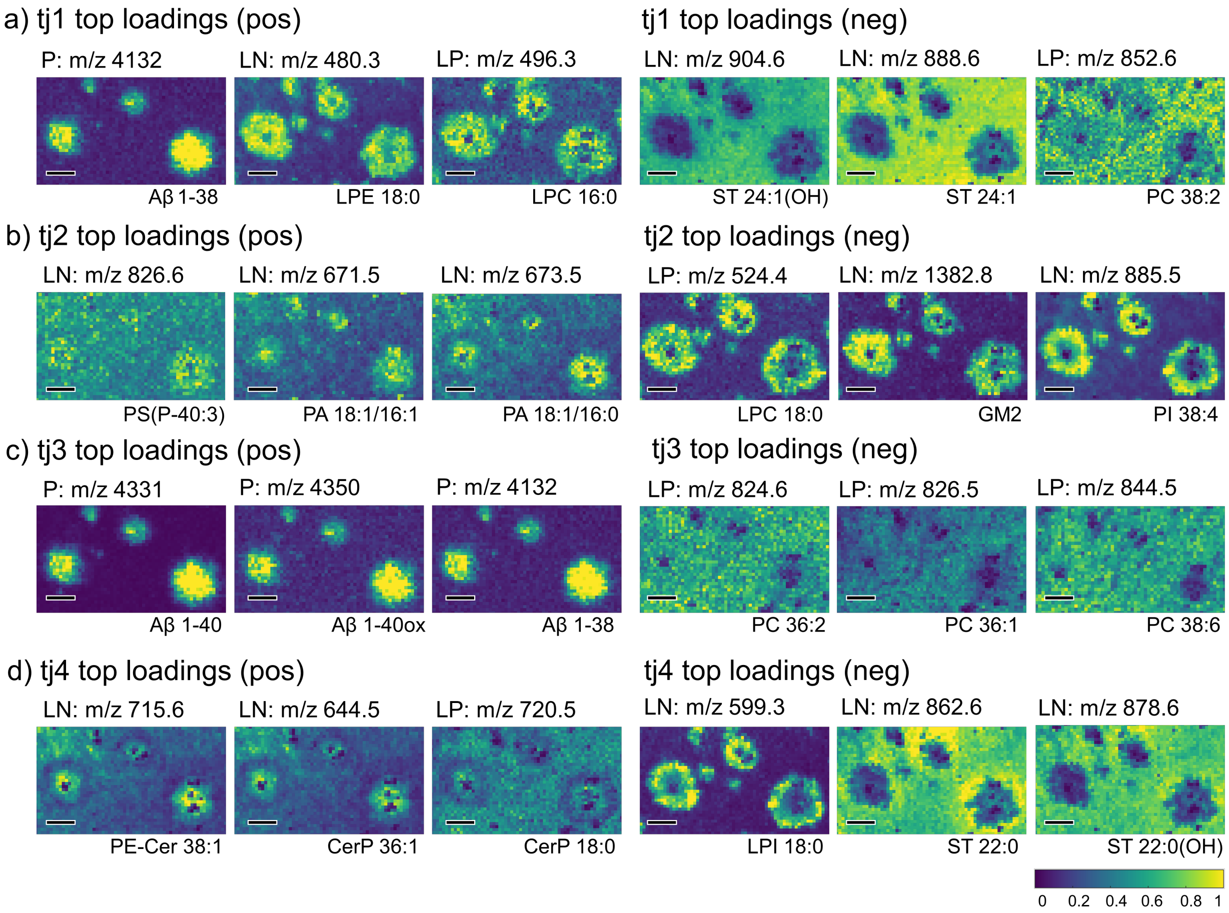

Supplemental Figure S4 Single ion images of top loadings from OnPLS modeling, tgSwe mouse brain tissue. Scale bar: 100 µm.

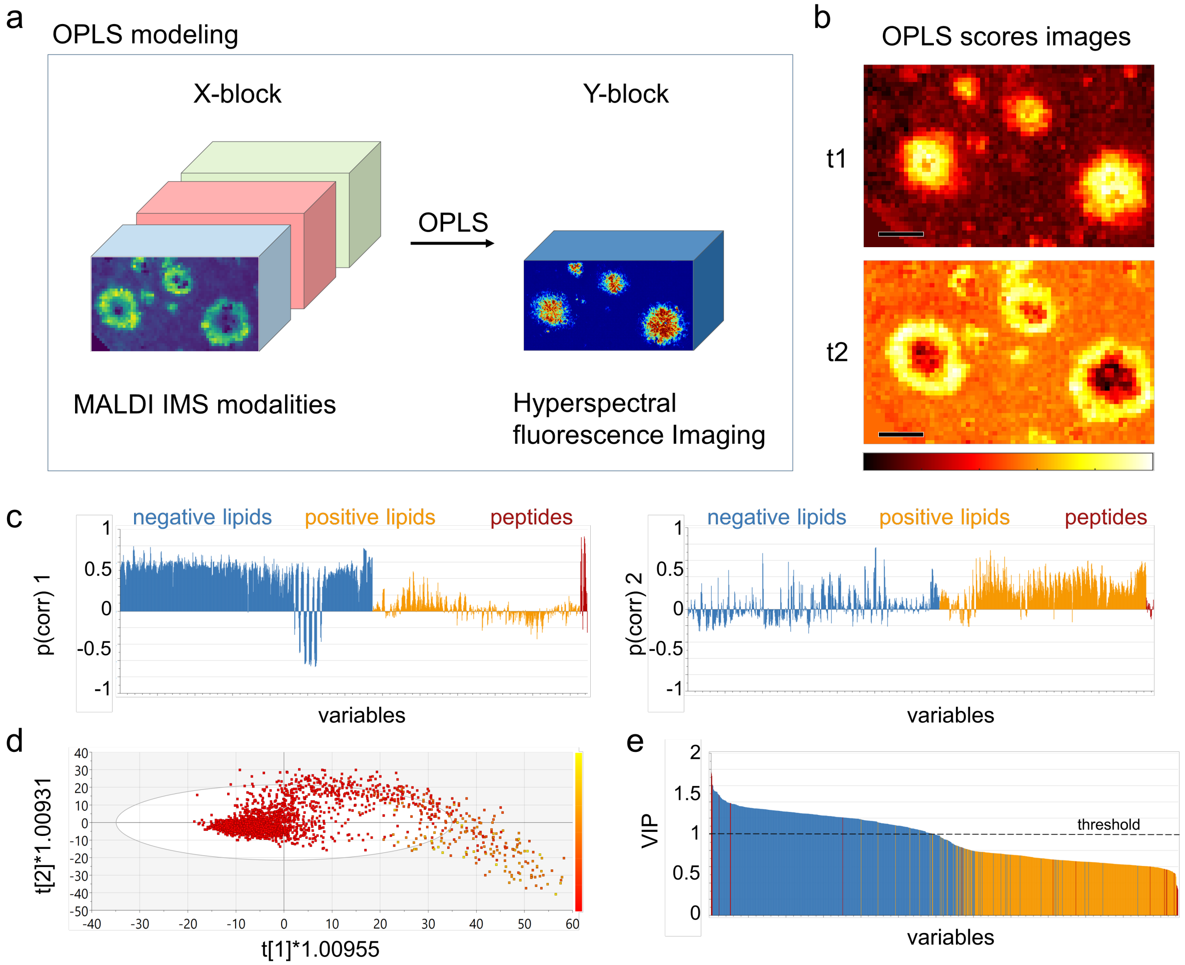

Supplemental Figure S5 OPLS modeling and image fusion of MSI[3]+ functional light microscopy (LC) data. a) OPLS modeling whereby registered MSI modalities are the x-block and imaging data from hyperspectral fluorescence microscopy are the Y-block. b) OPLS scores images, R^2Y 0.59, Q^2Y 0.57, Scale bar: 100 µm. c) loadings corresponding to scores images d) classic OPLS scores plot, ellipse 95 confidence region, coloration according to channel 10 e) VIP plot, sorted after VIP value, cut off at VIP=1.

A previously presented approach for data-driven fusion3 in-corporates automated means of filtering large number of variables (i.e. ion species) and is restricted to single mode MSI data and histological staining [3]. By passing pre-selected variables from VIP selection to the fusion protocol as demonstrated in our approach decreased the computation time from days to hours. Fusion computation of not VIP-filtered dataset was aborted after 6 days without completion. Using the VIP-filtered dataset generated fusion results after 6 hours of computation time (Microscopy dataset size: 1341x2029 pixels, 27 variables; IMS dataset size: 36x64 pixels, 2492 variables; IMS dataset size (reduced): 36x64 pixels, 1202 VIP variables; run on Intel(R) Core(TM) i7-7700K, CPU @ 4.2GHz, 32GB RAM).

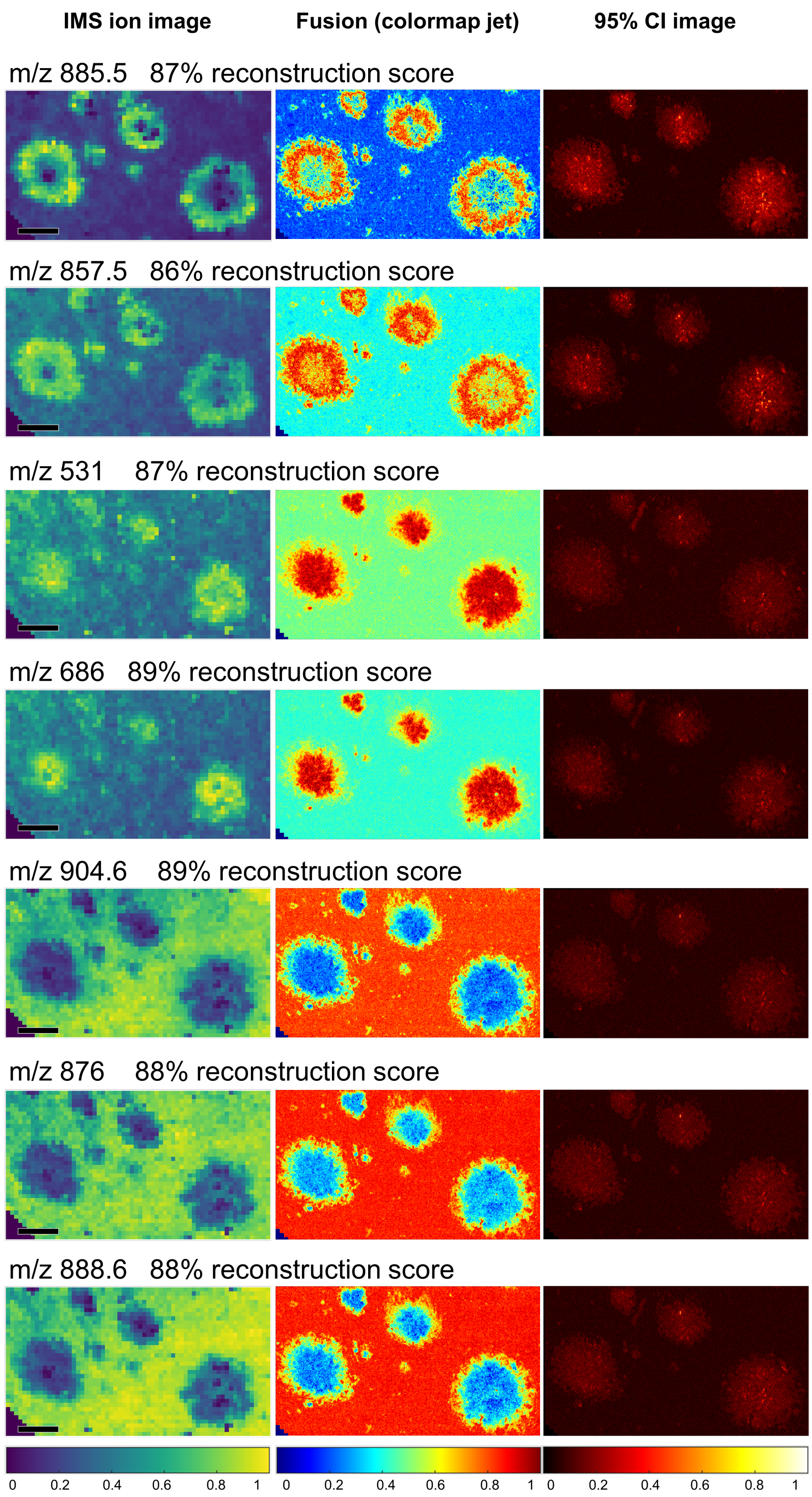

Supplemental Figure S6 Image data fusion predictions for negative ion mode lipids showing Aβ plaques in tgSwe mouse brain tissue. Visualization of 95% confidence intervals (CI) displaying confidence in the prediction in each pixel. Scale bar: 100 μm.

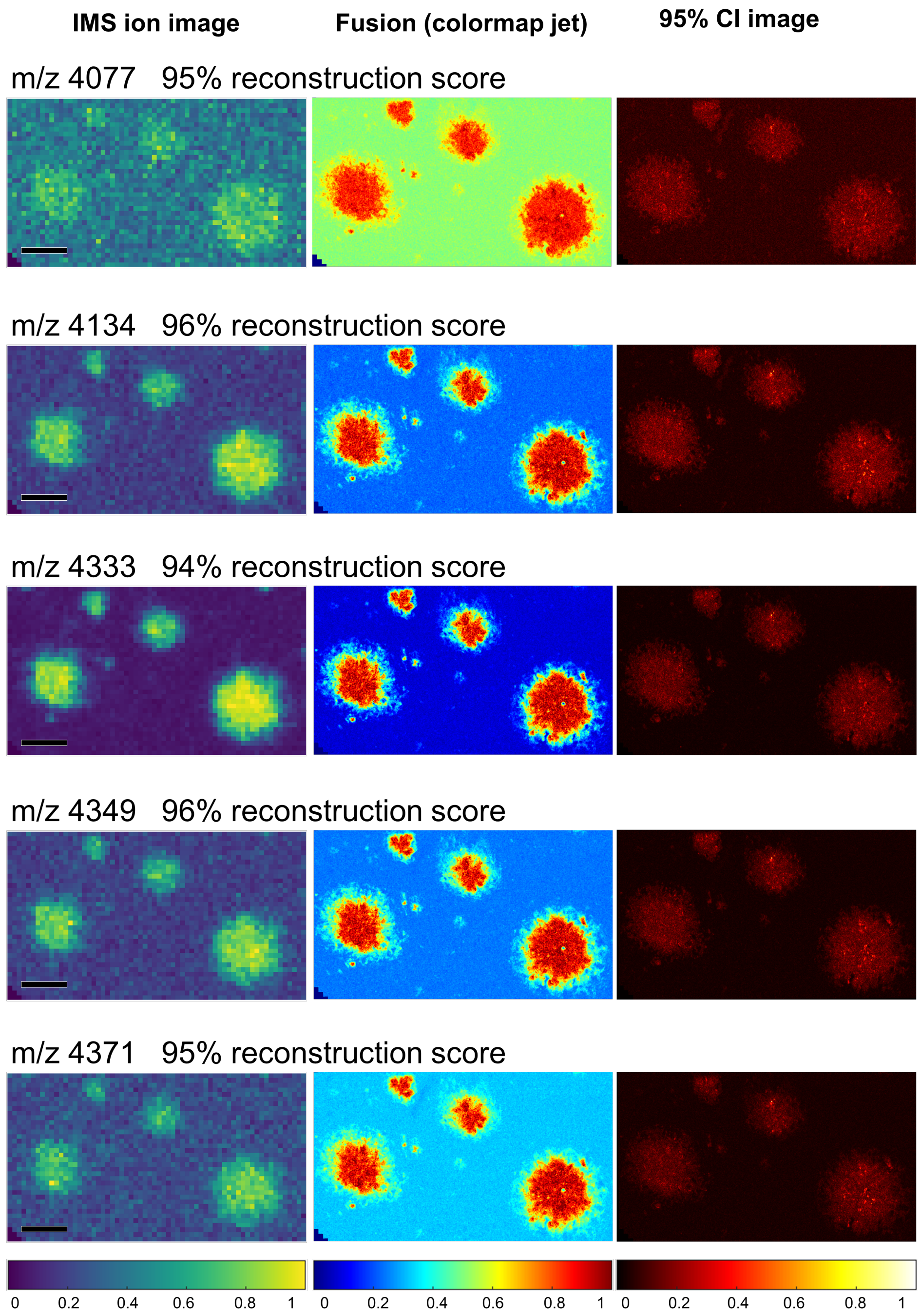

Supplemental Figure S7 Image data fusion predictions for Aβ peptides in tgSwe mouse brain tissue. Visualization of 95% confidence intervals (CI) displaying confidence in the prediction in each pixel. Scale bar: 100 µm.

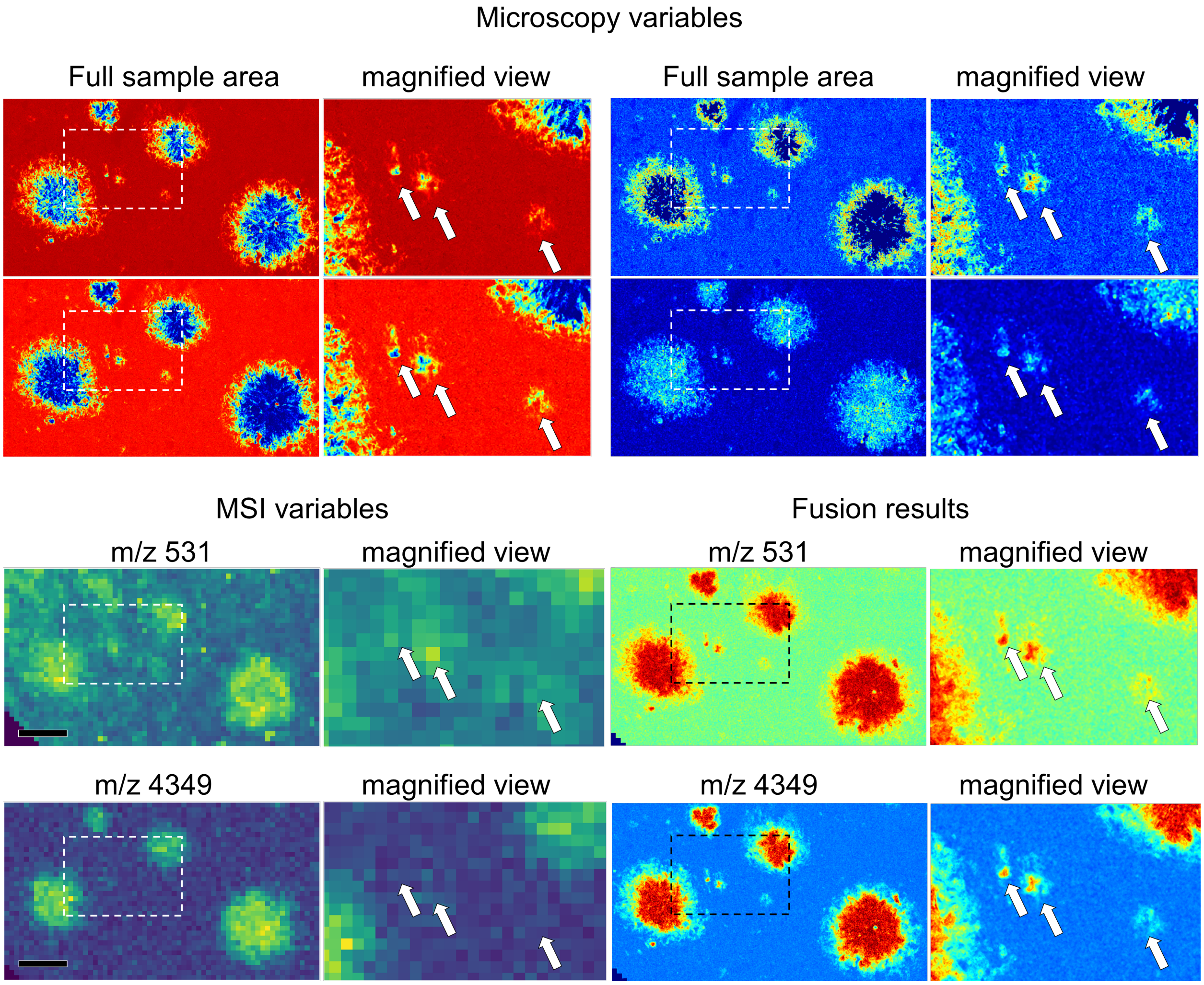

Supplemental Figure S8 Fusion prediction of small features that evade MSI detection. Even very small features that can be detected by microscopy but evade detection by MSI due to limitations in MSI spatial resolution or that are below the limit of MSI detection can be included in fusion predictions as a result of their hyperspectral signatures and their scoring in the regression model.

The 27 hyperspectral microscopy channels are subjected to 47 color space transformations[3] resulting in a total of 1296 microscopy modality images. The four microscopy images shown here are examples thereof to illustrate the how different structural details are captured. Here, particular focus is put on the small plaques annotated with arrows as some of these small plaques evade MSI detection. However, due to their detailed detection by hyperspectral microscopy, ion distributions can be predicted even for those small features as shown in fusion results.

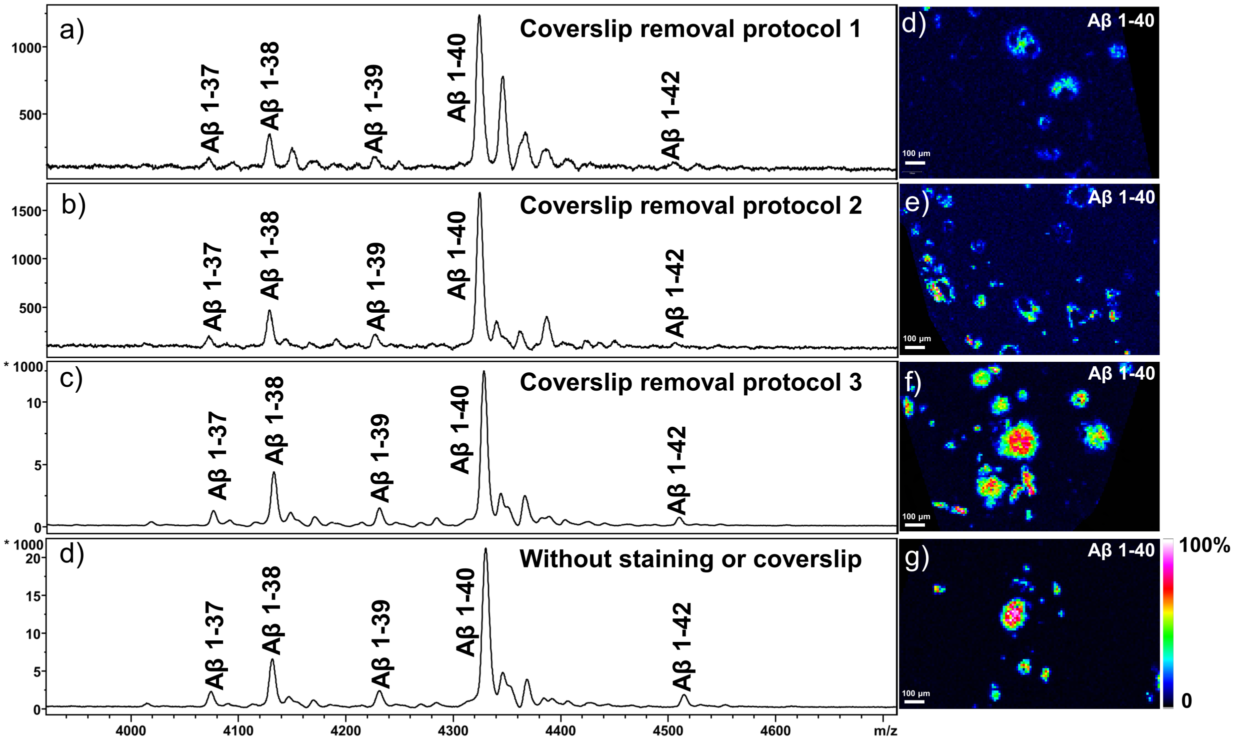

Supplemental Figure S9 Comparison of coverslip removal protocols. IMS was performed on tgSwe mouse brain tissue after different coverslip removal protocols had been applied, mass spectra (a-d) and single ion images of Aβ 1-40 (d-g) are shown. Coverslip removal protocol 1 and protocol 2 are not sufficient in removing coverslip mounting medium which is reflected in the spectral quality including signal intensity (a,b). Coverslip removal protocol 3 regains the signal quality and is on par with untreated tissue (c,d). Scale bars 100µm.

<u>Coverslip removal protocol 1 (insufficient):</u>

Coverslips were removed by soaking the glass slides in water for 18 hours at room temperature (23°C). Then tissue sections were washed with water for 30s, and dried under vacuum, followed by MALDI MSI peptide analysis.

<u>Coverslip removal protocol 2 (insufficient):</u>

Coverslips were removed by soaking the glass slides in water for 24 hours at room temperature (23°C). Tissue sections were then subjected to sequential washes in water for 1 minute, and EtOH for 30 seconds, and dried under vacuum before moving on to MALDI MSI peptide analysis.

<u>Coverslip removal protocol 3 (used in this study):</u>

Coverslips were removed by soaking the glass slides in water for 24 hours at room temperature (23°C). Tissue sections were then subjected to sequential washes in water for 8 minutes, 70% EtOH for 60 seconds, and EtOH for 30 seconds, and dried under vacuum before moving on to MALDI MSI peptide analysis.

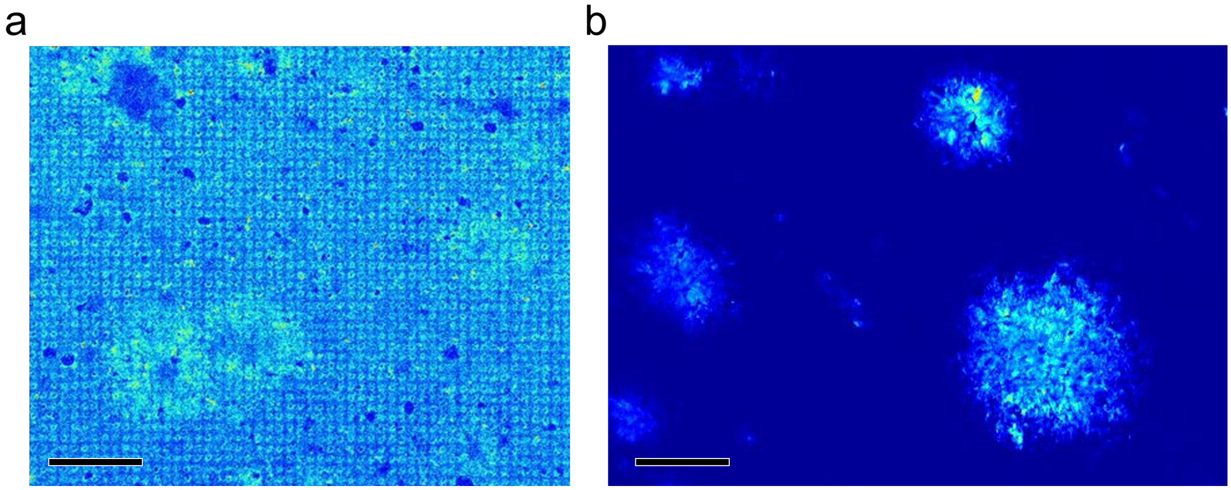

Supplemental Figure S10 Fluorescence microscopy images of LCO stained tgSwe mouse brain tissue before (a) and after (b) sample preparation optimizations. Scale bar 100µm.

3. Supplementary Table S1 (please find attached as separate xls file)

Example Data of MSI3 analysis showing the 20 most prominent variables for all MSI modalities and across both datasets. The comparison of the independent repeats shows very good overlap for the 15 most intense peaks: negative ion mode lipid modality 100%, positive ion mode lipid modality 93%, protein modality 86%. All images were generated in Matlab and individually color-scaled from 0:1, no other contrast or brightness adjustments were made. #Peak annotation was done by accurate mass matching to values reported in the literature [4]. *detected among the Top15 in dataset 1, **detected in the top15 in dataset 2.

4. References

1. Rohlfing, T., Image similarity and tissue overlaps as surrogates for image registration accuracy: widely used but unreliable. *IEEE transactions on medical imaging* **2012,** *31* (2), 153-163.
2. Russakoff, D. B.; Tomasi, C.; Rohlfing, T.; Maurer, C. R. In *Image Similarity Using Mutual Information of Regions*, Berlin, Heidelberg, Springer Berlin Heidelberg: Berlin, Heidelberg, 2004; pp 596-607.
3. Van de Plas, R.; Yang, J.; Spraggins, J.; Caprioli, R. M., Fusion of mass spectrometry and microscopy: a multi-modality paradigm for molecular tissue mapping. *Nature methods* **2015,** *12* (4), 366-372.
4. Michno, W.; Wehrli, P. M.; Koutarapu, S.; Marsching, C.; Minta, K.; Ge, J.; Meyer, S. W.; Zetterberg, H.; Blennow, K.; Henkel, C.; Oetjen, J.; Hopf, C.; Hanrieder, J., Structural amyloid plaque polymorphism is associated with distinct lipid accumulations revealed by trapped ion mobility mass spectrometry imaging. *J Neurochem* **2022,** *160* (4), 482-498.
